# Information Quality for Residency Applicants in Fellowship and Residency Electronic Interactive Database (FREIDA) and Program Websites

**DOI:** 10.7759/cureus.13900

**Published:** 2021-03-15

**Authors:** Shelbie D Kirkendoll, J. Bryan Carmody, Erika T Rhone

**Affiliations:** 1 General Surgery, Baystate Medical Center, Springfield, USA; 2 Pediatrics, Eastern Virginia Medical School, Norfolk, USA

**Keywords:** medical education, residency application, osteopathic medical graduate, international medical graduate, residency applicant, usmle, comlex, nrmp match, pediatric residency, freida

## Abstract

Background

Today’s residency applicants submit more applications than those in the past. To facilitate holistic review, many program directors have encouraged applicants to submit fewer applications. However, whether programs provide sufficient information to help applicants determine where to apply is unclear.

Objective

To evaluate the frequency of missing information on residency program websites and in the Fellowship and Residency Electronic Interactive Database (FREIDA).

Methods

We used FREIDA to identify all categorical pediatric residency programs in the United States. We noted the presence of information programs reported in each FREIDA data field. We compared information available on the program website for consistency with the information in FREIDA and additionally searched for current resident information and any description of the qualities of applicants/residents desired on the program website.

Results

Two hundred and eleven pediatric residency programs were included in FREIDA. Approximately 25% of programs did not include basic information such as number of first year residents, salary, work hours, or consideration of applicants requiring work visas. Over half of programs did not report minimum licensing examination scores required for interview consideration. Discrepancies between information on program websites and FREIDA related to work visas occurred in 6-8% of programs. While 88% of program websites included information on current residents, only 17% included any description of the applicant attributes sought by the program.

Conclusions

Many pediatric residency programs do not provide much of the information that applicants need to help determine if a program is a good fit or whether their application is competitive.

## Introduction

Residency applicants today submit many more applications than those in the past [1-3]. This is expensive for applicants, burdensome for residency program directors, and does not improve overall Match rates [1,4-8]. However, for applicants to sensibly apply to fewer programs, they must have access to high-quality information to help them select programs at which their application will be competitive.

To help applicants identify residency programs for which they may be a good fit, the American Medical Association (AMA) publishes the Fellowship and Residency Electronic Interactive Database Access (FREIDA). FREIDA allows applicants to search programs by specialty and geographic region and reports a variety of program-specific information. Anecdotally, most residency applicants seek information on programs both from FREIDA and individual program websites when determining where to apply. However, to our knowledge, the completeness of these sources of information has never been evaluated. Without an understanding of whether their application will be competitive - or rejected out of hand - applicants cannot sensibly limit the applications they submit. Therefore, we sought to evaluate the quality of information available to applicants on FREIDA and on pediatric residency program websites.

## Materials and methods

We reviewed information available in FREIDA and on individual residency program websites for all categorical pediatric residency programs in the United States. We chose to evaluate pediatric programs because this specialty attracts a large and diverse group of applicants, including many from osteopathic (DO) and international (IMG) medical schools. Data were collected in a cross-sectional fashion in May 2020.

We identified pediatric residency programs using FREIDA. FREIDA is a publicly available online residency database sponsored by the AMA. Each year, FREIDA surveys all Accreditation Council for Graduate Medical Education (ACGME)-accredited residency programs in the United States regarding their application requirements, demographics, and program amenities. Information obtained from this survey is uploaded to the database in February, August, and October. In addition to the annual survey, programs can update their individual profiles throughout the year upon request. Newly accredited programs are added throughout the year as they receive accreditation [9].

From FREIDA, we collected the information available to residency applicants featured in Table 1. After collecting data from FREIDA, we attempted to access the residency program’s website using the link available on each program’s FREIDA page. When no link was provided, or when the provided link did not work, we used standard search engines to locate the residency program’s website.

**Table 1:** Data Available in FREIDA FREIDA: Fellowship and Residency Electronic Interactive Database

Data Available in FREIDA	
Factor	Definitions
Geographic location	
Type of program:	
University-based	Training experience takes place in a hospital that serves as a primary affiliate of the medical school
Community-based/university affiliated	Training experience is in a community-based hospital that is affiliated with an academic medical center but is not a primary affiliate of the academic medical center
Community-based	Training experience takes place in a community setting that is not in an academic medical center or in a hospital with a medical school affiliation
Military	
Number of residents per entering class	
Name of program director	
Sponsorship of work visas	J-1 and H-1B
Minimum scores required for interview consideration for the United States Medical Licensing Examination (USMLE) Step 1 and 2 CK	
Minimum scores required for interview consideration for the Comprehensive Osteopathic Medical Licensing Examination (COMLEX-USA) Level 1 and 2 exams	
Mean scores for USMLE Step 1 and COMLEX-USA Level 1 of current residents/fellows	
Mean number of hours worked each week during the first program year	
Presence of a night float system	Defined as a rotation where residents only work during the nights with minimal or no daytime duties
Availability of special training opportunities:	
Rural track	Defined by a separate path solely devoted to rural primary care medicine
International rotation availability	
Research electives	Defined as a research rotation occurring while training during the program
Types of educational backgrounds of current residents:	Calculated as a 3-year average; does not include Canadian medical graduates.
Medical Doctor (MD)	
Doctor of Osteopathic Medicine (DO)	
International Medical Graduate (IMG)	

We then reviewed each program’s webpage to collect additional data to supplement and compare against the data found in FREIDA. Using information provided from the program’s website, we noted the names of program leadership; resident salary information; the percentage of residency graduates who pursue subspecialty fellowship training after graduation; minimum United States Medical Licensing Examination (USMLE) and Comprehensive Osteopathic Medical Licensing Examination (COMLEX-USA) scores required for interview consideration; and the number of letters of recommendation required. We also noted whether the program provided any information about their current residents, including resident names, photographs, and/or biographical information (medical school attended, hometown, personal biography, etc.). In addition, we searched for any description provided by the program illustrating the qualities of applicants/residents desired.

Regarding osteopathic residency applicants, we evaluated program websites to determine whether applications from DO students or graduates were accepted, and whether USMLE scores were required for DO applicants to be considered. Similarly, we searched program websites to determine if applications were accepted from IMGs and whether J-1 and/or H-1B work visas were accepted. We also noted any statements regarding specific USMLE score minimums for IMG applicants or the need for U.S. clinical experience for interview consideration. To determine whether a program had filled in the National Residency Matching Program (NRMP) Match, we used data from the 2020 NRMP Main Residency Match report [10].

We calculated descriptive statistics and determined the association between variables and outcomes of interest using chi-square tests or logistic regression, as appropriate. All statistical analyses were performed using IBM SPSS version 25 (Armonk, NY), with p < 0.05 considered significant.

## Results

Two hundred and eleven categorical pediatric residency programs were included in FREIDA. Of these, 109 (51.7%) were academic, 82 (38.9%) community/academic, 14 (6.6%) community, and six (2.8%) military programs. Seventy programs (32.7%) were located in the Northeast, with 70 (33.2%) in the South, 45 (21.3%) in the Midwest, and 27 (12.8%) in the West. Among the programs that participated in the NRMP Match in 2020, 175/197 (88.8%) filled all available resident positions. Program websites were identified and reviewed for 204 (96.6%) programs, while seven programs (3.3%) either did not have a program website available online via publicly available search engines and/or had a non-functional website link listed on FREIDA.

The number of programs with missing information on FREIDA and program websites varied, as shown in Table 2. There was no association between individual categories of missing data or the number of missing data categories on FREIDA/program websites and whether a program was filled or unfilled in the 2020 NRMP Match.

**Table 2:** Frequency of Missing Information in FREIDA and on Program Websites COMLEX-USA - Comprehensive Osteopathic Medical Licensing Examination; DO - doctor of osteopathic medicine; FREIDA - Fellowship and Residency Electronic Interactive Database; IMG - international medical graduate; MD - medical doctor; PGY-1 - postgraduate year 1; USMLE - United States Medical Licensing Examination

Factor	Number of Programs Missing Information	%
FREIDA (n = 211)		
Number of PGY-1 residents	53	25.1%
Salary information	55	26.1%
Number of letters of recommendation required for applicants	54	25.6%
Minimum USMLE Step 1 score required for interview consideration	87	41.2%
USMLE Step 1 score range for current residents	94	44.5%
Minimum COMLEX-USA Level 1 score required for interview consideration	119	56.4%
COMLEX-USA Level 1 score range for current residents	122	57.8%
Number/percentage of MD, DO, and IMG residents in program	82	38.9%
Accepts J-1 visa	54	25.6%
Sponsors H-1B visa	54	25.6%
Average hours worked per week	54	25.6%
Presence of night float system	54	25.6%
Program website link	11	5.2%
Program websites (n = 204)		
Number of letters of recommendation required	52	25.5%
Current resident information (e.g., names, photos, biographical profiles)	24	11.8%
Fellowship placement information	149	73.0%
Salary/benefit information	62	30.4%

Some programs had discrepancies between the information listed on FREIDA and the information found on their website. For instance, 6/204 (2.9%) programs had different program directors listed between the websites. Only 108 programs (51.2%) listed their willingness to interview applicants who required a J-1 or H-1B visa on both FREIDA and their program website. However, among these programs, nine (8.3%) provided discrepant information regarding applicants with a J-1 visa, while six (5.6%) provided discrepant information regarding applicants with H-1B visa.

Only 109 programs (53.4%) noted on their program website whether USMLE scores were required for DO applicants. Of these, 14 required DO applicants to submit USMLE scores, while 95 specifically noted that they did not. Similarly, although 137 programs (64.9%) reported considering applicants with J-1 visas on FREIDA, only 86 (62.8%) of these programs included information on whether U.S. clinical experience was required of applicants on their program website. Among these, 40 required U.S. clinical experience and 29 reported that U.S. clinical experience was preferred, while 20 noted no U.S. clinical experience requirement. Only 31 program websites (15.2%) included any statement regarding USMLE minimum scores or requirements (such as passing the clinical skills exam on the first attempt) for IMGs.

Most program websites (180/204; 88.2%) contained information on current residents. Resident photographs were provided by 159 (77.9%) of programs, and biographical information was provided by 164 (80.4%) programs. However, only a minority of program websites (35/204; 17.1%) included a written description of applicant qualities or attributes sought by the program.

## Discussion

Here, we present data on the amount of information available to pediatric residency applicants on FREIDA and program websites. Although most programs provided high-quality information to applicants, approximately 25% of programs failed to include important factors that may influence an applicant’s decision whether to apply to that program, such as screening thresholds for USMLE scores or willingness to evaluate applicants who require work visas.

In 2020, the average fourth year medical student from an MD school in the United States who applied to pediatric residency programs submitted 34 applications, while the average IMG submitted 44 [11]. While this number is lower than most other specialties, it nonetheless imposes a substantial burden on program directors [1,3]. Moreover, the overall Match rate for pediatric applicants is high: 99% of graduating MD students who only ranked pediatric residency programs successfully matched in 2020 [10]. It seems probable that most applicants to pediatric residency programs could submit fewer applications and enjoy the same likelihood of Match success. In fact, data from the NRMP’s Charting Outcomes in the Match reports show that allopathic seniors who rank just 5 programs have a better than 90% probability of matching [12]; osteopathic seniors who rank 7 programs [13] and IMGs who rank just 8 programs [14] have a similarly high likelihood of success in the Match. Accordingly, the Association of Pediatric Program Directors recommended that allopathic medical students with average Step 1 USMLE scores (216-234) apply to only 15 programs and osteopathic medical students with Step 1 USMLE scores (>220) apply to only 16 programs for the 2020-2021 residency application season [15].

The data presented here highlight one significant challenge that must be overcome for applicants to be convinced to apply to a lesser number of residency programs: inadequate information. “Overapplication” is expensive for applicants, but the cost of going unmatched is immeasurably greater. When applicants do not know where their application might be favorably received, applying broadly is a rational strategy. To make an informed decision requires universally available, high-quality data.

What is most striking about our findings is how frequently basic information is missing from FREIDA and program websites. Many programs do not report simple application requirements that may be used as exclusionary criteria. For instance, while many programs listed minimum USMLE scores for consideration, over half did not. It is possible that some of these programs do not use USMLE scores for screening and left this field blank intentionally to communicate this policy. However, any program that uses strict score filters but does not report them to applicants likely encourages students to apply whose applications will never be seriously reviewed. Similarly, while most programs communicated their willingness (or unwillingness) to consider IMGs who require work visas, one-quarter were silent on this issue. Many programs that invited J-1 visa applicants to apply still failed to mention other factors often used in application screening such as the need for U.S. clinical experience or IMG-specific USMLE scores.

We also identified important discrepancies between FREIDA data and residency program website data. Though these discrepancies occurred relatively infrequently, their importance should not be discounted. Similar to the adage about the man with two clocks who is never sure of the time, the presence of differing information on FREIDA and program websites generates uncertainty for applicants. It is also noteworthy that we struggled to locate some program websites; despite significant effort, we were ultimately unsuccessful in identifying a program website in 3.3% of cases. From the standpoint of an applicant, if the marginal cost of obtaining more information is less than the cost of one additional ERAS application, it is reasonable for applicants to apply to programs where their application may not even be considered, “just in case.”

The 2020-2021 interview season was conducted virtually due to the novel coronavirus pandemic. Initial signs suggest that virtual interviews may persist even after the pandemic is over, making it even more important for programs to have a high-quality website so applicants can learn about the program and its unique goals and mission. Currently, only a minority of programs provide a verbal description of the type of applicant they seek. This represents a clear opportunity for improvement. However, in this study, there was no relationship between the amount of information a program provided and the likelihood of that program filling in the Match, suggesting that there may be little penalty to programs with missing information in FREIDA or on their website. In the absence of a natural incentive to provide information to applicants, assistance from a third-party like the Accreditation Council for Graduate Medical Education may be needed. Conditioning the residency program's accreditation upon reporting a certain amount of standardized information to applicants would add an administrative burden to programs, but this cost may be outweighed by the benefits to applicants and programs alike by reducing excess applications.

At a minimum, we believe all programs should be required to transparently report exclusionary criteria. When applicants who do not meet the program’s minimum requirements nonetheless apply, it adds congestion to the system and is wasteful for the applicant and the program alike.

The strength of this work is its novelty. We are unaware of any previous systematic evaluation of FREIDA and pediatric program websites. This work therefore both adds a unique perspective and builds upon other surveys documenting applicant dissatisfaction with program websites. For instance, a 2008 survey of anesthesiology applicants found that only 2% of applicants felt that the majority of program websites contained all important content; the average program website contained only 46% of the content identified as important by applicants [16]. Similarly, 81% of respondents to a 2016 survey of otolaryngology applicants reported that online program information was insufficient [17]. Additionally, the analyses presented here are simple, straightforward, and lead to actionable conclusions.

Nonetheless, this study has several notable limitations. First, we evaluated only categorical pediatric programs. These findings may not be generalizable to other specialties. Second, we evaluated only two data sources - FREIDA and program websites - through which applicants obtain information. Applicants may obtain missing information using other resources which were not evaluated in this study. Third, our findings provide only a “snapshot” of the information available to residency applicants. It is likely that some of the data has changed since our review, and some programs may have corrected the discrepancies and deficiencies we identified. Finally, it is possible that some of the information for which we searched was in fact available in areas of the program's website that were not easily located. However, we thoroughly evaluated each website, and if we were unable to locate the information, we believe that many (if not most) applicants would have been similarly unsuccessful.

## Conclusions

In a cross-sectional review of pediatric residency program websites and profiles on FREIDA, important deficiencies were found in at least 25% of programs. To assist applicants in selecting residency programs at which their application will be seriously considered, efforts should be made to report consistent, standardized information to potential applicants.

## References

[1] Carmody JB, Rosman IS, Carlson JC (2021). Application fever: reviewing the causes, costs, and cures for residency application inflation. Cureus.

[2] Hammoud MM, Andrews J, Skochelak SE (2020). Improving the residency application and selection process: an optional early result acceptance program. JAMA.

[3] Liao NN, Mahan JD, Scherzer R (2020). The pediatric match frenzy: an overview and an approach for mentoring medical students. Acad Pediatr.

[4] Berger JS, Cioletti A (2016). Viewpoint from 2 graduate medical education deans: application overload in the residency match process. J Grad Med Educ.

[5] Blackshaw AM, Watson SC, Bush JS (2017). The cost and burden of the residency match in emergency medicine. West J Emerg Med.

[6] Moore DB (2018). Not a cheap investment: estimating the cost of the 2017 to 2018 ophthalmology residency match to the applicant and program. J Acad Ophthalmol.

[7] Nikonow TN, Lyon TD, Jackman SV, Averch TD (2015). Survey of applicant experience and cost in the urology match: opportunities for reform. J Urol.

[8] Weissbart SJ, Kim SJ, Feinn RS, Stock JA (2015). Relationship between the number of residency applications and the yearly Match rate: time to start thinking about an application limit?. J Grad Med Educ.

[9] (2020). FREIDA, the AMA residency & fellowship database - FAQs & glossary. https://assets.ama-assn.org/resources/doc/freida/x-pub/freida-glossary.pdf.

[10] (2020). National Resident Matching Program: results and data - 2020 main residency match. https://mk0nrmp3oyqui6wqfm.kinstacdn.com/wp-content/uploads/2020/06/MM_Results_and-Data_2020-1.pdf.

[11] (2020). ERAS statistics: preliminary data (ERAS 2020) - residency. https://www.aamc.org/eras-statistics-2019.

[12] (2020). National Resident Matching Program: charting outcomes in the match - U.S. allopathic seniors. https://mk0nrmp3oyqui6wqfm.kinstacdn.com/wp-content/uploads/2019/10/Charting-Outcomes-in-the-Match-2018_Seniors-1.pdf.

[13] (2020). National Resident Matching Program: charting outcomes in the match - seniors students of U.S. osteopathic medical schools. Osteopathic Medical Schools.

[14] (2020). National Resident Matching Program: charting outcomes in the match - international medical graduates. https://mk0nrmp3oyqui6wqfm.kinstacdn.com/wp-content/uploads/2018/06/Charting-Outcomes-in-the-Match-2018-IMGs.pdf.

[15] (2020). APPD/COMSEP/AMSPDC letter to our pediatrics community. https://mk0pediatricpro0yafp.kinstacdn.com/wp-content/uploads/2020/06/Improving_Pediatric_Residency_Application_ProcessRecommendations.pdf.

[16] Chu LF, Young CA, Zamora AK, Lowe D, Hoang DB, Pearl RG, Macario A (2011). Self-reported information needs of anesthesia residency applicants and analysis of applicant-related web sites resources at 131 United States training programs. Anesth Analg.

[17] Ward M, Pingree C, Laury AM, Bowe SN (2017). Applicant perspectives on the otolaryngology residency application process. JAMA Otolaryngol Head Neck Surg.

